# Novel Challenges and Therapeutic Options for Pulmonary Embolism and Deep Vein Thrombosis

**DOI:** 10.3390/jpm14080885

**Published:** 2024-08-21

**Authors:** Chiara Cavallino, Marco Franzino, Mohamed Abdirashid, Ludovica Maltese, Elodi Bacci, Francesco Rametta, Fabrizio Ugo

**Affiliations:** Cardiology Division, Sant’Andrea Hostpital, 13100 Vercelli, Italy; marco.franzino@outlook.it (M.F.); mohamed.abdirashid@aslvc.piemonte.it (M.A.); ludovica.maltese@aslvc.piemonte.it (L.M.); elodi.bacci@aslvc.piemonte.it (E.B.); francesco.rametta@aslvc.piemonte.it (F.R.); fabrizio.ugo@aslvc.piemonte.it (F.U.)

**Keywords:** acute pulmonary embolism, deep vein thrombosis, systemic fibrinolysis, percutaneous treatment, catheter-directed thrombolysis, aspiration thrombectomy, ultrasound-assisted thrombolysis, bleeding

## Abstract

Acute pulmonary embolism (PE), often resulting from deep vein thrombosis (DVT), is the third most frequent cause of cardiovascular death and is associated with increasing incidence, causing considerable morbidity and mortality. This review aims to evaluate the efficacy, safety, and outcomes of treatment options in the management of acute PE and DVT, encompassing both established and emerging technologies, such as catheter-directed thrombolysis, aspiration thrombectomy, and other endovascular techniques. A comprehensive literature review was conducted, assessing clinical studies, trials, and case reports that detail the use of percutaneous interventions for PE and DVT and analyzing the advantages and disadvantages of each percutaneous system. Several percutaneous treatments have shown promising results, especially in cases where rapid thrombus resolution is critical, such as in high- and intermediate–high-risk patients. The incidence of major complications, such as bleeding, remains a consideration, though it is generally manageable with proper patient selection and technique. It is fundamentally important to tailor the specific treatment strategy to the clinical and anatomical characteristics of each patient. Percutaneous treatments for acute PE and DVT represent valuable options in the therapeutic arsenal, offering enhanced outcomes in appropriately selected patients. Ongoing advancements in technology and technique, along with comprehensive clinical trials, are essential to further define the role and optimize the use of these interventions.

## 1. Introduction

Pulmonary embolism (PE), the third most frequent cause of cardiovascular death, is characterized by a steadily increasing incidence, which correlates with the aging population and the rise in neoplastic pathology prevalence [1,2,3,4,5,6]. Pulmonary embolism accounts for approximately one million deaths worldwide annually and imposes a significant healthcare burden, costing nearly EUR 4 billion in Europe and USD 19 billion in the US each year [1,7]. Frequently, PE results from the embolization of a deep vein thrombosis (DVT) in the pulmonary vasculature: DVT is identified in 70% of PE patients through venography and in 30–50% through compression ultrasonography (CUS) [8,9,10,11].

These two conditions share the same risk factors and often occur together, requiring simultaneous evaluation and management. Virchow’s triad of hypercoagulability, venous stasis, and endothelial injury represents common risk factors, which can be categorized into genetic factors—including thrombophilia (factor V Leiden mutation, prothrombin gene mutation, protein C deficiency, protein S deficiency, and hyperhomocysteinemia)—and acquired factors, such as prolonged immobilization, malignancy, recent orthopedic surgery, indwelling venous catheters, obesity, pregnancy, smoking, and the use of oral contraceptives. The management of PE involves addressing five treatment goals: ensuring hemodynamic stability, restoring perfusion, facilitating tissue oxygenation, minimizing therapy-related complications, and preventing disease recurrence. In this review, we explore various therapeutic strategies ranging from well-established approaches to innovative interventions.

## 2. Current Guidelines/Consensus

The 2019 ESC guidelines defined four risk indicators that, when combined, stratify patients into high-, intermediate–high-, intermediate–low-, and low-risk categories (Table 1). Each risk class delineates a distinct therapeutic pathway, ranging from systemic thrombolysis to anticoagulant therapy alone, with various percutaneous or surgical mechanical techniques in between [12].

Following the release of the 2019 ESC guidelines on PE, several consensus documents have been published, including the ESC consensus document on percutaneous treatment options for acute pulmonary embolism in 2022, which has expanded the boundaries of use of the most innovative percutaneous reperfusion techniques [13]. Conversely, current vascular surgery guidelines and the ESC consensus document about the treatment of deep vein thrombosis limited the application of percutaneous treatment in a limited clinical context [14,15].

Therefore, based on current scientific knowledge, standard-dose systemic fibrinolysis should be the therapy of choice for high-risk patients. The use of lower dosages should be limited to sporadic cases where thrombolysis is not contraindicated, but the risk of bleeding is considered clinically relevant based on clinical experience.

## 3. Therapy

### 3.1. Pulmonary Embolism—Systemic Fibrinolysis

Systemic fibrinolysis (plasminogen activator (rt-PA), urokinase, or streptokinase) [16,17] is the first strategy in high-risk patients, defined by haemodynamic instability, and it should be an option in intermediate-risk patients that show signs of clinical deterioration (Table 2) [12,18]. This approach has a demonstrated ability to reduce all-cause mortality and PE-related mortality and recurrence compared with unfractionated heparin alone [19,20]. However, it is associated with a notable risk of bleeding: A meta-analysis of 16 randomized controlled trials (RCTs) involving 2057 patients revealed a number needed to treat (NNT) of 59 for all-cause mortality, while the number needed to harm (NNH), represented by major bleeding events, was 18 [19]. In an effort to mitigate the risk of bleeding, various trials and observational studies have explored the use of lower doses of thrombolytics [21,22,23,24]. Nonetheless, reduced-dose thrombolysis is currently not recommended because the results of these trials and meta-analysis do not demonstrate functional improvements equivalent to those achieved with full-dose thrombolysis. A meta-analysis of five studies comprising a total of 440 patients demonstrated that low-dose rt-PA was associated with no significant difference in bleeding rates compared to standard-dose rt-PA [21]. Furthermore, there was no observed significant difference in the risk of major bleeding events, recurrent rt-PA use, or all-cause death when comparing low-dose rt-PA with the use of heparin [21].

### 3.2. Pulmonary Embolism—Percutaneous Options

The use of catheter-directed therapies in PE should be considered when thrombolysis has either failed or is contraindicated when patients at intermediate–high risk do not improve despite anticoagulant therapy after 24–48 h or experience hemodynamic or respiratory deterioration (Table 3, Table 4, Table 5 and Table 6) [12,13].

#### 3.2.1. Mechanical Fragmentation

Mechanical thrombus fragmentation with a rotational pigtail catheter, despite lacking supporting evidence, is widely available and technically relatively easy to perform; therefore, even in 2021, in Europe, it was still the most commonly used catheter-based therapy for acute PE [44].

This technique can be modified by rotating the pigtail catheter around a guidewire exiting from a side-hole [45], combining this technique with the downsizing of peripheral balloon inflation [18] or thrombolytic administration.

Several over-the-wire rotational thrombectomy systems that combine a motor-driven, rotating, flexible sinusoidal tip for mechanical clot fragmentation with active negative pressure to remove debris were developed, but these techniques are unappetizing because of the poor literature and higher risk of vascular wall injury [46,47,48].

#### 3.2.2. Rheolytic Thrombectomy

The AngioJet device (Boston Scientific) is a rheolytic system that combines the disruption of blood clots with pressurized fluid with the active removal of debris through a vacuum; furthermore, this device allows for the local administration of thrombolytic drugs with the Power Pulse option [49,50,51].

Despite demonstrating effectiveness with improvements in the RV-to-LV diameter ratio and pulmonary arterial pressures, particularly in high- and intermediate–high-risk PE cases [32,33], the AngioJet device has been associated with a notable rate of complications.

In a meta-analysis of 35 studies conducted by Kuo et al., the findings revealed several adverse events, including five procedure-related deaths, bradyarrhythmia, heart block, hemoglobinuria, temporary renal insufficiency, hemoptysis, and five major hemorrhages [34].

Moreover, the U.S. Food and Drug Administration’s Manufacturer (FDA) and User Facility Device Experience database has documented several additional deaths related to the device, leading to the issuance of a “black-box” warning on the device label by the FDA.

#### 3.2.3. Catheter-Directed Thrombolysis (CDT)

The cornerstone and earliest form of percutaneous treatment is catheter-directed thrombolysis (CDT), which involves delivering a low-dose thrombolytic drug via a catheter directly into the thrombotic pulmonary artery. This technique can be performed with different types of catheters, and the most used types are the pigtail and dedicated side-hole catheter, with the latter often inherited from acute limb ischemia treatment [52,53].

Among dedicated side-hole catheters, the Cragg–McNamara catheter [Medtronic] has been extensively studied in trials. Kroupa et al. demonstrated, despite a small sample size and short observation period, a significant reduction in the RV-to-LV ratio (*p* = 0.03) and a significant decrease in systolic pulmonary artery pressure (PAP) by ≥30% (*p* = 0.001) in the CDT group compared to the anticoagulation group in intermediate-risk PE patients, with no intracranial or life-threatening bleeding in either group [25].

In the CANARY trial, which was underpowered and prematurely interrupted due to the COVID-19 pandemic after enrolling 94 out of 288 planned patients, the CDT group (12 mg alteplase in unilateral PE or 24 mg alteplase in bilateral PE) showed a significantly lower mean RV-to-LV ratio compared to the anticoagulation group (*p* = 0.01) and more frequent RV recovery at 3 months after CDT (*p* = 0.009). Notably, one case of BARC-type 3a major bleeding (non-fatal gastrointestinal bleeding) occurred in the CDT group, with no instances of intracranial or fatal bleeding in either group [26].

The Uni-Fuse catheter [AngioDynamics] is another option approved by the European Union (EU) for PE treatment. However, the Fountain Infusion System by Merit Medical and the Pulse-Spray Infusion System by AngioDynamics are not yet approved by the European Union (EU).

All approved dedicated side-hole catheters have a diameter of 4–5 Fr, and thrombolytic dosing regimens vary between centers. Typically, alteplase infusion ranges from 0.5 to 1.0 mg/h in one or both main pulmonary arteries for up to 24 h, without exceeding the total dosage of 30 mg [13,54,55].

#### 3.2.4. Ultrasound-Assisted Thrombolysis

Ultrasound-assisted thrombolysis (USAT) represents an evolution of CDT, combining pharmacological thrombolysis with high-frequency ultrasound energy (2 MHz) and causing the disaggregation of uncross-linked fibrin fibers, an increase in the surface area of the thrombus, and the optimization of the penetration and action of fibrinolytic drugs [56,57,58].

The EkoSonic Endovascular System [Boston Scientific] is a two-lumen catheter and is the only device currently approved for performing USAT that has been extensively tested in several trials.

The ULTIMA trial demonstrated the efficacy and safety of USAT (using 10–20 mg rt-PA over 15 h) in intermediate-risk PE, with significant reductions in the mean RV-to-LV ratio at 24 h (*p* < 0.001) and mean PAP within 12 h (*p* < 0.001) without increasing bleeding risk compared to systemic anticoagulation alone [39].

The OPTALYSE PE trial demonstrated that various dose and timing strategies of thrombolytic therapy (rt-PA doses of 4 mg, 6 mg, or 12 mg per pulmonary artery, with infusion durations of 2, 4, or 6 h) in intermediate-risk PE were associated with an increased RV-to-LV ratio and low thrombotic burden, with no increase in the bleeding risk [28].

Conversely, the SEATTLE II trial showed a decrease in the mean RV-to-LV ratio from baseline to 48 h post-procedure (*p* < 0.0001) and a decrease in the mean pulmonary artery systolic pressure in high-risk or intermediate–high-risk individuals, administering a cumulative rt-PA dose of 24 mg, albeit with a higher incidence of major bleeding (10%) [29].

In the SUNSET sPE trial, both USAT and CDT (Uni-Fuse or Cragg–McNamara systems) showed a significant reduction in thrombus burden without significant differences between the two groups in intermediate–high-risk PE; however, the mean RV-to-LV ratio was more markedly decreased in the CDT group than in the USAT group (*p* < 0.01) [27].

A meta-analysis of 28 studies involving 1430 patients treated with USAT showed a mortality rate of 2.9% during their hospital stay and overall long-term mortality at the time of follow-up of 4.1%, with major bleeding complications occurring in 5.4% [30]. The prospective KNOCOUT PE registry showed even lower total bleeding rates within 72 h (1.6%) and all-cause mortality at 30 days (1.0%) in intermediate–high-risk or high-risk PE patients treated with a mean rt-PA dose of 18.1 mg and a mean infusion time of 10.5 h [31].

Two different French-size devices are available: 5.4 Fr for EkoSonic and 7.8 Fr for EkoSonic+ (characterized by 50% more ultrasound power and 32% more lysis); vascular access is also available via the femoral or jugular vein [55]. During USAT, the dose of heparin should be reduced: In some centers, the protocol provides an infusion of unfractionated heparin reduced to 500 IU/h, while in other centers, the heparin dosage targets an anti-Xa level of 0.5–0.7 IU/mL. For patients pretreated with low-molecular-weight heparin or direct oral anticoagulants within 8–12 h before thrombolysis infusion, the heparin bolus should be omitted. Instead, the heparin infusion should be started with an anti-Xa target of 0.3–0.5 IU/mL for the initial hours of thrombolysis [13].

#### 3.2.5. Aspiration Thrombectomy

Aspiration thrombectomy involves the removal of thrombi from the pulmonary artery through catheters that generate suction, through mechanical negative pressure pumps such as the Indigo Aspiration System [Penumbra], or by the manual creation of a vacuum using a syringe, as seen in the FlowTriever Retrieval/Aspiration System [Inari Medical].

The prospective, multicenter, single-arm FLARE study evaluated the safety and efficacy of the first-generation FlowTriever device in intermediate–high-risk PE, demonstrating a significant decrease in the RV-to-LV ratio after an average follow-up of 48 h and a low incidence of major bleeding (1%) [35].

The ongoing, multicenter, prospective FLASH registry is investigating the safety and effectiveness of the second-generation FlowTriever device, showing a significant decrease in on-table mean PAP (*p* < 0.0001) and RV-to-LV ratio at 48 h after the procedure (*p* < 0.0001) in patients who are high risk (8%), intermediate–high risk (76%) and intermediate–low risk (16%), with a major adverse event rate of 1.8% at 48 h [36].

Interestingly, the FLAME trial, a non-randomized study, showed a significant reduction in the primary endpoint (an in-hospital composite of all-cause mortality, bailout to an alternate thrombus removal strategy, clinical deterioration, and major bleeding) in high-risk PE patients treated with the FlowTriever arm (17%) against patients treated with the context arm (63%), without differences between systemic thrombolytics (66.7%) and anticoagulation alone (71.4%); moreover, in-hospital mortality with respect to the FlowTriever and context groups occurred in 1.9% and 29.5% of high-risk PE patients, respectively [37].

Currently, there is limited literature on the Indigo Aspiration System; however, the prospective, single-arm, multicenter EXTRACT-PE trial showed a significant decrease in RV-to-LV ratio by 0.43 ± 0.26 (*p* < 0.0001) and a significant reduction in systolic PAP of 4.3 mmHg (*p* < 0.0001) within 48 h in intermediate-risk PE using the 8 Fr Indigo Aspiration System, with a major adverse event rate of 1.7% [38].

The main differences between these aspiration systems lie in the mode of aspiration, the rigidity of the catheter, and the device’s dimensions. The Indigo Aspiration System includes a 12 Fr and 16 Fr aspiration catheter, a pump providing a vacuum suction system equipped with the new computer-aided Lightning system, and a separator wire with a soft tip used to disrupt the thrombus and facilitate aspiration.

On the other hand, the FlowTriever device offers a choice between three aspiration catheters of varying diameters (16, 20, and 24 Fr), each equipped with a 60 mL aspiration syringe. Additionally, it provides three catheters with self-expanding nitinol mesh disks (from 6 mm to 25 mm in diameter) used in approximately 25% of procedures when the simple aspiration technique is unsuccessful [35]. This device is also equipped with FlowSaver, an external blood return system consisting of a 40 μm filtration system that holds back thrombi and returns blood to the patient.

### 3.3. Pulmonary Embolism—Other Strategy

One of the oldest but currently available revascularization strategies in acute pulmonary embolism is surgical embolectomy, which can be performed in both high-risk patients with thrombolysis contraindications or failure and in intermediate–high-risk PE with risk or signs of clinical or instrumental deterioration [12,59,60]. This strategy demonstrated a significant reduction in RV diameter and systolic PAP (*p* < 0.001) [61], with similar early and long-term survival against thrombolysis [62].

In chronic thromboembolic pulmonary hypertension (CTEPH), percutaneous pulmonary angioplasty represents a solid treatment option for selected patients with inoperable CTEPH or persistent/recurrent pulmonary hypertension (PH) after pulmonary endarterectomy (PEA). It improves hemodynamics, right heart function, and exercise capacity. This technique should be performed in high-volume CTEPH centers because it is associated with serious complications, some of which may be fatal [63,64].

For patients at high risk of venous thromboembolism (VTE) with contraindications relative to anticoagulant therapy or those experiencing recurrent PE despite adequate anticoagulation, vena cava interruption should be considered. This entails the insertion of a mechanical filter device that obstructs the passage of venous clots into pulmonary circulation [12]. The PREPIC trial, the only randomized controlled trial on inferior vena cava (IVC) filters, demonstrated short-term and long-term advantages in preventing pulmonary embolism recurrence in patients receiving IVC filters. However, it failed to show survival benefits when using IVC filters and revealed a significant increase in DVT recurrence in the IVC filter group [65].

### 3.4. Deep Vein Thrombosis—Percutaneous Options

Patients with extensive lower extremity DVT, despite treatment with anticoagulant therapy, face a high risk of developing post-thrombotic syndrome (PTS), occurring in 25% to 75% of cases despite anticoagulant therapy [65,66,67]. To mitigate this risk, various devices and techniques have been explored, including catheter-directed thrombolysis (CDT), ultrasound-assisted thrombolysis (USAT), the AngioJet device, the Trellis device, balloon venoplasty, stent implantation, and manual aspiration.

Key randomized trials in this area include TORPEDO, CaVenT, ATTRACT, and CAVA. The TORPEDO trial demonstrated the superiority of percutaneous endovenous intervention plus anticoagulation over anticoagulation alone in reducing venous thromboembolism (VTE) and PTS at 6 months in symptomatic patients with proximal DVT [40]. Conversely, the ATTRACT trial did not show a difference in PTS and recurrent VTE over 24 months between anticoagulation alone and CDT or CDT combined with AngioJet/Trellis-8 in patients with femoral or more proximal DVT, although the use of AngioJet/Trellis-8 led to more major bleeding within 10 days [42]. A pre-specified iliofemoral subgroup analysis of the trial demonstrated a reduced PTS severity score in patients treated with CDT combined with Angiojet/Trellis-8 without increasing major bleeding rates [68].

The CaVenT trial showed a significant absolute risk reduction (14.4%) of PTS in patients with symptomatic iliofemoral DVT treated with CDT with rtPA in addition to anticoagulant therapy against anticoagulant treatment alone at 24 months [41], with an increase in absolute risk reduction to 28% after 5 years of follow-up [67]. The CAVA trial compared the use of USAT vs. standard anticoagulation therapy alone in patients with iliofemoral DVT, showing no differences between the two treatment groups in PTS after 12 months [43].

To summarize, a meta-analysis of these four trials (TORPEDO, CaVenT, ATTRACT, and CAVA) demonstrated that early thrombus removal techniques are more effective than anticoagulation alone in preventing any PTS (*p* = 0.05) and, mostly, moderate to severe PTS (*p* < 0.001) at the price of a significantly increased risk of major bleeding (*p* = 0.02) [15]. Based on these studies, guidelines suggest considering early thrombus removal strategies in selected patients with symptomatic iliofemoral DVT, particularly patients with the highest risk of developing PTS (extensive clot burden involving the iliofemoral level), high chances of technical success (recent thrombus within two weeks of onset), and low bleeding risks [15,69]. Conversely, in DVT limited to femoral, popliteal, or calf veins, early thrombus removal is not recommended [15].

Additionally, percutaneous intervention may be indicated in cases of phlegmasia cerulea dolens, a condition associated with high mortality rates (20–40%), particularly if gangrene is present [15,70,71]. In upper extremity DVT, systemic thrombolysis has been replaced by CDT, although early thrombus removal is generally contraindicated in most patients with symptomatic primary upper extremity DVT according to guidelines [15].

Stent implantation in DVT remains a controversial topic, with no direct comparison trials between stenting and no stenting after early thrombus removal in acute DVT. Stent placement is preferred for iliac vein obstruction or inferior vena cava obstruction, especially in chronic intrinsic or extrinsic stenosis, like May–Thurner syndrome [72]. Contrarily, balloon angioplasty alone is preferred for flow-limiting femoropopliteal venous lesions [73,74]. To summarize, percutaneous options for treating deep vein thrombosis (DVT) should be considered in patients without a high bleeding risk and who are affected by recent femoro-popliteal thrombosis to reduce the risk of post-thrombotic syndrome. Conversely, DVT distal to the iliofemoral segment should not be treated invasively. In the absence of trials comparing different technologies, any of the mentioned devices can be used, with the choice depending on the experience and availability of the center.

## 4. Ongoing Studies

Various interesting studies are currently underway to consolidate knowledge regarding the use of these devices in patients with intermediate- and intermediate–high-risk PE and to clarify some doubts and controversial results from previous trials.

The PRAGUE-26 trial will enroll 558 patients with intermediate–high-risk acute PE. Patients will be randomized in a 1:1 ratio to CDT or relative to standard anticoagulation therapy, and the primary outcome is a clinical composite of all-cause mortality, PE recurrence, or cardiorespiratory decompensation within 7 days of randomization.

The USAT is being retrospectively investigated in the HI-PEITHO trials, which are enrolling 406 patients. This trial aims to compare the 7-day incidences of all-cause death, hemodynamic decompensation, and PE recurrence using USAT plus heparin versus standard anticoagulation alone. Furthermore, the STRATIFY trial will compare USAT with low-dose systemic thrombolysis and standard anticoagulation alone. This single-blind, phase 3 trial aims to assess a reduction in the computed tomography Miller score at 96 h in 210 intermediate–high-risk PE patients. It could be intriguing to develop a trial capable of confirming or refuting the unexpected results of the underpowered SUNSET sPE trial, particularly regarding the significant difference in the RV-to-LV ratio in favor of CDT.

The STORM-PE trial, a randomized, multicenter study, will compare the reduction in RV-to-LV ratio in intermediate–high-risk PE patients treated with the recent Indigo Aspiration System 12 Fr catheter against standard anticoagulation therapy. Another ongoing phase 1 trial (ClinicalTrials.gov: NCT05612854) is investigating the safety and efficacy of the Indigo Aspiration System 8 Fr, CDT, or pigtail mechanical fragmentation compared to standard anticoagulation alone in 200 patients with intermediate–high-risk PE.

The FlowTriever system will be investigated in two noteworthy studies. The PEERLESS trial will compare the FlowTriever system with CDT in 550 intermediate–high-risk patients. The primary endpoint will be a composite of clinical outcomes, including all-cause mortality, intracranial hemorrhage, and major bleeding, constructed as a hierarchical win ratio at hospital discharge or 7 days post-procedure.

In the PEERLESS II trial, 1200 intermediate-risk PE patients will be randomized to receive FlowTriever treatment or a standard anticoagulation protocol. The study aims to assess a composite clinical endpoint constructed as a win ratio, with a hierarchy of hemodynamic decompensation and all-cause hospital readmission at 30 days, and 3-month all-cause death, PE-related mortality, and major bleeding.

Another intriguing ongoing trial is the PE-TRACT trial, a phase 3, assessor-blinded study designed to evaluate the short-term safety and efficacy of catheter-directed therapies (CDT or mechanical thrombectomy) compared to standard anticoagulation protocols in 500 patients with sub-massive PE, proximal pulmonary artery thrombus, and right ventricular dilation.

We hope that these studies will yield exciting results in terms of efficacy and reassuring results in terms of safety, allowing these devices to become an additional tool in our armamentarium to improve both short- and long-term outcomes in patients suffering from a pulmonary embolism at intermediate risk and above.

In the realm of percutaneous treatment for deep vein thrombosis (DVT), several trials are ongoing, focusing on different devices and procedural approaches. The DEFIANCE trial aims to assess the efficacy of the ClotTriever System [Inari Medical] compared to anticoagulation therapy alone in achieving and maintaining vessel patency in subjects with symptomatic unilateral iliofemoral DVT. The SPADE trial seeks to determine the efficacy of the AngioJet catheter in achieving angiographic luminal patency at the procedure’s conclusion without requiring adjuvant CDT in iliofemoral DVT.

Lastly, two trials (ClinicalTrials.gov: NCT06124768 and NCT05286710) are exploring the advantages of utilizing either ipsilateral distal calf venous access or contralateral femoral access compared to the standard ipsilateral popliteal access for the percutaneous treatment of iliofemoral DVT.

## 5. Conclusions

The landscape of percutaneous therapies for pulmonary embolism and deep vein thrombosis is continuously evolving, with a plethora of technologies now available to clinicians. While these advancements offer promising avenues for improving patient outcomes, the evidence supporting their efficacy and safety remains variable. Therefore, the need for further randomized controlled trials (RCTs) is paramount to establish robust guidelines and refine treatment protocols.

As highlighted in this review, the selection of the most appropriate device or combination of devices should be tailored to the individual patient, considering factors such as thrombus burden, comorbidities, bleeding risk, and procedural feasibility. By synthesizing the current knowledge and providing insights into the comparative effectiveness of different interventions, this review serves as a valuable resource for clinicians navigating the complex landscape of PE management.

More specifically, in high-risk PE, when thrombolysis is contraindicated or has failed, we suggest using an aspiration thrombectomy system to rapidly reduce thrombus burden and right ventricle afterload. In intermediate–high-risk PE, the range of usable devices expands. We considered using catheter-directed thrombolysis, ultrasound-assisted thrombolysis, or aspiration thrombectomy, carefully balancing the risks and benefits of each technique on a case-by-case basis.

Although not investigated in any trial, we consider the combination of different percutaneous systems, such as an aspiration system combined with catheter-directed thrombolysis, to be a viable strategy in both moderate–high- and high-risk cases. This approach is particularly valuable when the initial percutaneous system has not been sufficiently effective or when the thrombotic burden is very high.

In conclusion, while percutaneous therapies hold great promise in the management of PE and DPT, continued research, innovation, and collaboration between clinicians, interventional cardiologists and radiologists, researchers, and the industry are imperative to unlock their full potential and address the unmet needs of patients with this life-threatening condition.

## Figures and Tables

**Table 1:** Acute pulmonary embolism: risk stratification according to ESC Guidelines [12]. CTPA: Computed tomography pulmonary angiography; PE: pulmonary embolism; TTE: transthoracic echocardiogram.

Class of PE Risk		Indicators of Risk			
		Haemodynamic Instability	Clinical Parameters of PE Severity and/or Comorbidity	Right Ventricular Dysfunction (TTE or CTPA)	Elevated Levels of Cardiac Troponins
High		+	(+)	+	(+)
Intermediate	Intermediate–high	-	+	+	+
	Intermediate–low	-	+	One (or none) positive	
Low		-	-	-	-

The colors in the table represent the risk levels: Red indicates high risk, Orange indicates intermediate risk, and Green indicates low risk.

**Table 2:** Signs and symptoms of clinical deterioration: (+) increase; (−) reduction; ECMO: extracorporeal membrane oxygenation; PE: pulmonary embolism; RA: right atrium; RV: right ventricle; TAPSE: tricuspid annular plane systolic excursion.

Parameters/Signs of Deterioration in PE			
Clinical	Laboratory	Echocardiography	Need for
Blood pressure (−)	Serum lactate (+)	Pulmonary arterial pressure (−)	Mechanical ventilation
Heart rate (+)	Serum HS troponin (+)	TAPSE (−)	ECMO
Respiratory rate (+)	Serum natriuretic peptide (+)	RV/RA gradient > 60 mmHg	Vasopressors or inotropes
Diuresis (−)	Serum creatinine (+)	Pulmonary ejection acceleration time < 60 ms	
Blood oxygen saturation (−)	Serum liver enzyme (+)		
Consciousness (−)			

**Table 3:** European Union-approved device for acute pulmonary embolism. F: Femoral; Fr: French; J: jugular.

Mechanism of Thrombus Removal	Device (Manufacturer)	Vascular Access	Technical Aspect	Literature
Mechanical fragmentation	Pigtail	F/J	Diameter: 6–8 Fr	/
Thrombolytic infusion	Cragg-McNamara (Medtronic, Minneapolis, MN, USA)	F	Diameters: 4 and 5 Fr Lengths: 40, 65, 100, and 135 cm	Kroupa [25], CANARY [26], SUSET-sPE [27]
	Uni-Fuse (AngioDynamic, Latham, NY, USA)	F	Diameters: 4 and 5 Fr Lengths: 45, 90, and 135 cm compatible with a 0.035″ guidewire	SUNSET-sPE [27]
Ultrasound-assisted thrombolysis (USAT)	EkoSonic (Boston Scientific, Marlborough, MA, USA)	F/J	Diameters: 5.4 and 7.8 Fr Lengths: 106 and 135 cm	ULTIMA, OPTALYSE PE [28], SEATTLE II [29], SUNSET-sPE [27], Pei [30], KNOCOUT PE [31]
Rheolytic thrombectomy	AngioJet (Boston Scientific)	F/J	Plus the option of thrombolytic injections (Power Pulse). Diameters: 6 and 8 Fr Length: 120 cm	Akbal [32], Li [33], Kuo [34]
Aspiration with the option of mechanical fragmentation	FlowTriever (Inari Medical, Irvine, CA, USA)	F	Diameters: 16, 20, and 24 Fr Lengths: 90 and 113 cm Catheter sizes: S (6–10 mm), M (11–14 mm), L (15–18 mm), XL (19–25 mm)	FLARE [35], FLASH [36], FLAME [37]
Aspiration with mechanical fragmentation	Indigo (Penumbra, Alameda, CA, USA)	F/J	Diameters: 12 and 16 Fr Lengths: 80, 100 and 115 cm	EXTRACT PE [38]

**Table 4:** Current trials. (*) Plus anticoagulation. CDT: Catheter-directed thrombosis; DVT: deep vein thrombosis; LV: left ventricle; PE: pulmonary embolism; PTS: post-thrombotic syndrome; RV: right ventricle; tPA: tissue plasminogen activator; USAT: ultrasound-accelerated thrombolysis; VTE: venous thromboembolism.

Trial	Year	Design	Device	Population	Sample	Treatment Arm	Control Arm	Efficacy Outcome	Safety Outcome
ULTIMA [39] (NCT01166997)	2013	Randomized, open-label	EkoSonic (Boston Scientific)	Intermediate risk PE	59	tPA-USAT (10 mg) *	Anticoagulation monotherapy	∆RV/LV ratio at 24 h: 0.30 ± 0.20 vs. 0.03 ± 0.16 (*p* < 0.001)	0 deaths, 0 major bleeds, 3 minor bleeds at 90 days
SEATTLE II [29] (NCT01513759)	2015	Single arm	EkoSonic (Boston Scientific)	Intermediate–high-risk PE	150	tPA-USAT (12–24 mg) *	/	∆RV/LV ratio at 48 h: 0.42 ± 0.36 (*p* < 0.0001)	7 deaths, 15 major bleeds at 30 days
OPTALYSE PE [28] (NCT02396758)	2018	Randomized, open-label	EkoSonic (Boston Scientific)	Intermediate risk PE	101	tPA-USAT (4 mg, 6 mg, or 12 mg) *	Arm 1 (4 mg/lung/2 h), arm 2 (4 mg/lung/4 h), arm 3 (6 mg/lung/6 h), and arm 4 (12 mg/lung/6 h)	RV/LV ratio reduced in all arms at 48 ± 6 h	5 major bleeds at 72 h
SUNSET sPE [27] (NCT02758574)	2021	Randomized, single-blind	EkoSonic (Boston Scientific) vs. Cragg-McNamara (Medtronic) or Uni-Fuse (AngioDynamics, Latham, NY, USA)	Intermediate risk PE	81	tPA-USAT (4–8 mg) *	Anticoagulation plus CDT	∆RV/LV ratio at 48 h: 0.37 ± 0.34 vs. 0.59 ± 0.42 (*p* = 0.01)	2 major bleeds, 3 minor bleeds, and 1 in-hospital death
Kroupa et al. [25] (pilot study)	2022	Randomized, open-label	Cragg-McNamara (Medtronic)	Intermediate–high-risk PE	23	CDT (Alteplase 20 mg) *	Anticoagulation monotherapy	∆RV/LV ratio at 48 h: 0.88 ± 0.16 vs. 1.42 ± 0.44 (*p* = 0.01)	0 BARC Type 5 or 3c major bleeds
CANARY [26] (NCT05172115)	2022	Randomized, open-label	Cragg-McNamara (Medtronic)	Intermediate–high-risk PE	94	CDT (Alteplase 12–24 mg) *	Anticoagulation monotherapy	∆RV/LV ratio reduced at 3 months: 0.7 vs. 0.8 (*p* = 0.01)	1 BARC Type 3a major bleed
FLARE [35] (NCT02692586)	2019	Single-arm	FlowTriever (Inari medical)	Intermediate risk PE	106	FlowTriever System *	/	∆RV/LV ratio at 48 h: 0.41 ± 0.05 (*p* < 0.0001)	All-cause death: 0; major bleeding: 0.9% at 48 h
EXTRACT-PE [38] (NCT03218566)	2021	Single-arm	Indigo (Penumbra)	Intermediate risk PE	119	Indigo System *	/	∆RV/LV ratio at 48 h: 0.43 ± 0.26 (*p* < 0.0001)	All-cause death: 1.1%; major bleeding: 1.6% at 48 h
FLAME [37] (NCT04795167)	2023	Prospective	FlowTriever (Inari medical)	High-risk PE	104	FlowTriever System *	Other therapies	Composite of all-cause mortality, clinical deterioration, bailout, and major bleeding: 17% vs. 63.9%	All-cause death: 1.9% vs. 29.5%; major bleeding: 11.3% vs. 24.6%
TORPEDO [40]	2010	Randomized, open-label	Angiojet, Trellis, venoplasty, stent	Symptomatic acute proximal DVT	183	Percutaneous endovenous intervention *	Anticoagulation monotherapy	Development of PTS at 6 months was significantly higher in the Control Group (27.2% vs. 3.4%, *p* < 0.001)	No significant difference in the rate of bleeding
CaVenT [41] (NCT00251771)	2012	Randomized, open-label	/	Symptomatic acute iliofemoral DVT	209	CDT with rtPA *	Anticoagulation monotherapy	Significant absolute risk reduction (14.4%) of PTS	20 bleeding CDT-related complications
ATTRACT [42] (NCT00790335)	2017	Randomized, open-label	AngioJet (Boston Scientific), Trellis (Covidien, Dublin, Ireland)	Acute femoral or more proximal DVT	692	Pharmacomechanical thrombolysis *	Anticoagulation monotherapy	No difference in PTS and recurrent VTE	More major bleeding with pharmacomechanical thrombolysis within 10 days
CAVA [43] (NCT00970619)	2021	Randomized, open-label	Ekos Endowave System (Ekos Corporation, South Bothell, WA, USA)	Acute iliofemoral DVT	184	USAT *	Anticoagulation monotherapy	No reduction in PTS	No significant bleeding difference

**Table 5:** Technical PROS and CONS of different percutaneous device catheters.

	Mechanical Fragmentation with Pig-Tail Catheter	Mechanical Fragmentation with Rotational Thrombectomy Systems (Cleaner, Rotarex, and Aspirex)	Rheolytic Thrombectomy (Angiojet)	Catheter-Directed Thrombolysis (Cragg–McNamara, Uni-Fuse)	Ultrasound-Assisted Thrombolysis (EkoSonic)	Aspiration Thrombectomy with the Option of Mechanical Fragmentation (FlowTriever)	Aspiration with Mechanical Fragmentation (Indigo)
PROS	Easy to perform. Cheap. Low risk of wall injury.	Easy to perform.	Combination of thrombus fragmentation and aspiration, optional thrombolytic drugs local infusion.	Low catheter diameter (4–5 Fr).	Combination of local pharmacological thrombolysis (low dose) with high-frequency ultrasound energy. Low catheter diameter (5.4 and 7.8 Fr).	Removal of high thrombotic burden in a short time. Optional combination of mechanical fragmentation. Possibility to filter and reinfuse blood.	Removal of high thrombotic burden in a short time. Combination of aspiration and mechanical fragmentation. More flexible and maneuverable catheter. Thrombus detection technology to limit blood loss.
CONS	Distal embolization. Poor literature.	Poor literature. Higher risk of vascular wall injury.	Higher rate of systemic complications and consequent “black-box” warning by the FDA.	Long duration of thrombolytic infusion (12–24 h).	Effectiveness of ultrasound is still not solidly demonstrated in the literature. Expensive. Mean infusion time 10–12 h.	High catheter diameter (16, 20, and 24 Fr). More rigid catheter.	High catheter diameter (12 and 16 Fr). No possibility to filter and reinfuse aspirated blood.

**Table 6:** Ongoing trials. (*) Plus anticoagulation. CDT: Catheter-directed therapy; DVT: deep vein thrombosis; PE: pulmonary embolism; USAT: ultrasound-accelerated thrombolysis.

Trial	Design	Population	Sample	Intervention	Control	Primary Outcomes
PRAGUE-26	Multicenter, randomized	Intermediate–high-risk PE	558	CDT *	Anticoagulation monotherapy	Clinical composite of all-cause mortality, PE recurrence, or cardiorespiratory decompensation
HI-PEITHO	Multicenter, randomized	Intermediate–high-risk PE	406	USAT *	Anticoagulation monotherapy	Composite of PE-related mortality, cardiorespiratory decompensation or collapse, or non-fatal symptomatic and objectively confirmed PE recurrence
STRATIFY	Multicenter, 1:1:1 randomized	Intermediate–high-risk PE	210	USAT or low-dose thrombolysis *	Anticoagulation monotherapy	Miller score
STORM-PE	Multicenter, randomized	Intermediate–high-risk PE	100	Aspiration embolectomy (Indigo system) *	Anticoagulation monotherapy	Reduction in RV-to-LV ratio
NCT05612854	Multicenter, randomized	Intermediate–high-risk PE	200	Indigo Aspiration System (8 Fr), CDT or pigtail mechanical fragmentation *	Anticoagulation monotherapy	MACE
PEERLESS	Multicenter, randomized	Intermediate–high-risk PE	550	Aspiration embolectomy (FlowTriever system) *	CDT *	All-cause death, intracranial hemorrhage, major bleeding, haemodynamic decompensation
PEERLESS II	Multicenter, randomized	Intermediate risk PE	1200	Aspiration embolectomy (FlowTriever system) *	Anticoagulation monotherapy	Haemodynamic decompensation, all-cause hospital readmission, bailout therapy
PE-TRACT	Multicenter, randomized	Sub-massive PE, proximal pulmonary artery thrombus, and right ventricular dilation	500	CDT or mechanical thrombectomy *	Anticoagulation monotherapy	Peak oxygen consumption, NYHA class, major bleeding
DEFIANCE	Multicenter, randomized	Symptomatic unilateral iliofemoral DVT	300	Mechanical thrombectomy (ClotTriever System) *	Anticoagulation monotherapy	Vessel patency rate
NCT06124768	Single-center, observational	Acute iliofemoral DVT	210	Mechanical Thrombectomy via ipsilateral deep calf venous access or contralateral femoral venous access	Mechanical thrombectomy via ipsilateral popliteal venous access	Vessel patency rate
NCT05286710	Single-center, randomized	Acute iliofemoral DVT	160	Mechanical Thrombectomy via distal calf venous access or contralateral femoral access	Mechanical thrombectomy via ipsilateral popliteal venous access	Incidence of post-thrombotic syndrome

## Data Availability

No new data were created or analyzed in this study. Data sharing is not applicable to this article.

## Abbreviations

CDT	catheter-directed thrombolysis
CTEPH	chronic thromboembolic pulmonary hypertension
DVT	deep vein thrombosis
EU	European Union
FDA	U.S. Food and Drug Administration
LV	left ventricle
PAP	pulmonary artery pressure
PE	pulmonary embolism
PTS	post-thrombotic syndrome
RCT	randomized controlled trial
rt-PA	recombinant tissue plasminogen activator
RV	right ventricle
USAT	ultrasound-assisted thrombolysis
VTE	venous thromboembolism
